# Comparative Genomics Analysis of Ciliates Provides Insights on the Evolutionary History Within “Nassophorea–Synhymenia–Phyllopharyngea” Assemblage

**DOI:** 10.3389/fmicb.2019.02819

**Published:** 2019-12-12

**Authors:** Bo Pan, Xiao Chen, Lina Hou, Qianqian Zhang, Zhishuai Qu, Alan Warren, Miao Miao

**Affiliations:** ^1^Institute of Evolution and Marine Biodiversity, Ocean University of China, Qingdao, China; ^2^Laboratory for Marine Biology and Biotechnology, Qingdao National Laboratory for Marine Science and Technology, Qingdao, China; ^3^Department of Genetics and Development, Columbia University Medical Center, New York, NY, United States; ^4^Savaid Medical School, University of Chinese Academy of Sciences, Beijing, China; ^5^Yantai Institute of Coastal Zone Research, Chinese Academy of Sciences, Yantai, China; ^6^Ecology Group, Technical University of Kaiserslautern, Kaiserslautern, Germany; ^7^Department of Life Sciences, Natural History Museum, London, United Kingdom

**Keywords:** ciliated protozoa, evolution, introns, phylogenetic relationship, stop codon usage

## Abstract

Ciliated protists (ciliates) are widely used for investigating evolution, mostly due to their successful radiation after their early evolutionary branching. In this study, we employed high-throughput sequencing technology to reveal the phylogenetic position of Synhymenia, as well as two classes Nassophorea and Phyllopharyngea, which have been a long-standing puzzle in the field of ciliate systematics and evolution. We obtained genomic and transcriptomic data from single cells of one synhymenian (*Chilodontopsis depressa*) and six other species of phyllopharyngeans (*Chilodochona* sp., *Dysteria derouxi*, *Hartmannula sinica*, *Trithigmostoma cucullulus*, *Trochilia petrani*, and *Trochilia* sp.). Phylogenomic analysis based on 157 orthologous genes comprising 173,835 amino acid residues revealed the affiliation of *C. depressa* within the class Phyllopharyngea, and the monophyly of Nassophorea, which strongly support the assignment of Synhymenia as a subclass within the class Phyllopharyngea. Comparative genomic analyses further revealed that *C. depressa* shares more orthologous genes with the class Nassophorea than with Phyllopharyngea, and the stop codon usage in *C. depressa* resembles that of Phyllopharyngea. Functional enrichment analysis demonstrated that biological pathways in *C. depressa* are more similar to Phyllopharyngea than Nassophorea. These results suggest that genomic and transcriptomic data can be used to provide insights into the evolutionary relationships within the “Nassophorea–Synhymenia–Phyllopharyngea” assemblage.

## Introduction

**FIGURE 1:**
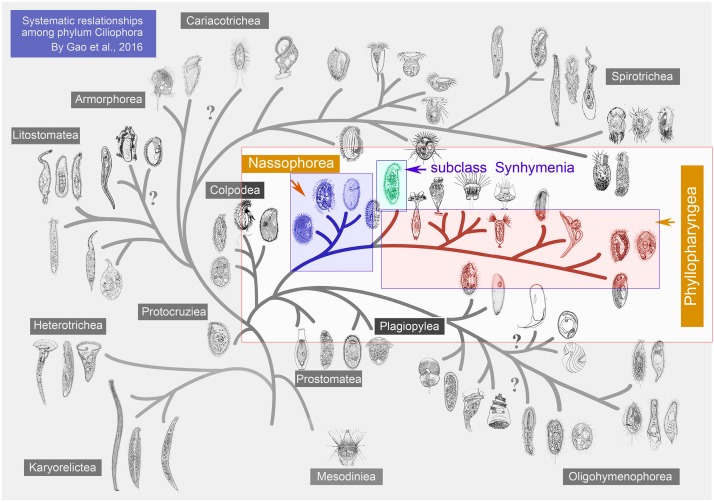
Hypothetical evolution of ciliated protozoa based on both morphological and molecular data to show the relationships and the positions of the taxa at class level. Synhymenia, Phyllopharyngea, and Nassophorea are highlighted. This figure was modified from Gao et al. (2016).

Members of the order Synhymeniida are characterized by a cytopharyngeal basket of well-developed nematodesmata bound proximally by the presence of a band of somatic dikinetid cilia (Kivimaki et al., 2009; Figure 1). Corliss assigned the Synhymeniida to the subclass Hypostomata and placed the nassulids and microthoracids in a separate order (Nassulida) within the same subclass (Corliss, 1979; Table 1). Subsequently, Synhymeniida was assigned to the class Nassophorea because it possesses a cytopharyngeal basket (nasse), a character shared by the typical nassophorean groups (nassulids and microthoracids) (Small and Lynn, 1985; Lynn and Small, 2002; Lynn, 2008; Table 1). However, phylogenetic studies based mainly on the small subunit ribosomal DNA (SSU rDNA) gene and other related genes (e.g., LSU rDNA, ITS, alpha-tubulin), as well as a comparison of ultrastructural synapomorphies and autapomorphies, suggested that the subclass Synhymenia should be moved from the class Nassophorea to Phyllopharyngea (Gong et al., 2009; Zhang et al., 2014; Gao et al., 2016; Table 1). Based on a study of the ultrastructure of the typical synhymenian species *Zosterodasys agamalievi* Kivimaki et al. (1997) concluded that the synhymenians cannot be assigned to either Phyllopharyngea or Nassophorea, because they have a combination of characters that precludes their inclusion in either. A recent study used a phylogenomic approach to test evolutionary relationships within the class Nassophorea, however, the dataset did not represent a broad sampling of all major ciliate clades (Lynn et al., 2018). Genomes of typical synhymeniids, for example, have yet to be reported (Lynn and Small, 2002).

**TABLE 1:** Taxonomic schemes for the classification of 10 species ciliates in four systematic schemes.

**Corliss, 1979**	**de Puytorac, 1994**	**Lynn, 2008**	**Adl et al., 2019**
Kinerofragminophora	Nassophorea	Nassophorea	Nassophorea
Hypostomata	Nassulina	Synhymeniida	Nassulida
Synhymeniida	Synhymeniida	Scaphidiodontidae	*Nassula*
*Chilodontopsis*	*Chilodontopsis*	*Chilodontopsis*	*Furgasonia*
Nassulida	Nassulida	Nassulida	Microthoracida
Nassulina	*Nassula*	Nassulidae	*Pseudomicrothorax*
Nassulidae	*Furgasonia*	*Nassula*	Phyllopharyngea
*Nassula*	Microthoracida	Furgasoniidae	Synhymeniida
Furgasoniidae	*Pseudomicrothorax*	*Furgasonia*	*Chilodontopsis*
*Furgasonia*	Phyllopharyngea	Microthoracida	Cyrtophoria
Microthoracina	Cyrtophoria	Leptopharyngidae	*Chilodonella*
Leptopharyngidae	Chilodonellida	*Pseudomicrothorax*	*Trithigmostoma*
*Pseudomicrothorax*	Chilodonellidae	Phyllopharyngea	*Dysteria*
Cyrtophorida	*Trithigmostoma*	Cyrtophoria	*Trochilia*
Chlamydodontina	*Chilodonella*	Chlamydodontida	*Hartmannula*
Chilodonellidae	Dysteriida	Chilodonellidae	Chonotrichia
*Chilodonella*	Hartmannulina	*Chilodonella*	*Chilodochona*
*Trithigmostoma*	Hartmannulidae	*Trithigmostoma*	
Dysteriina	*Hartmannula*	Dysteriida	
Hartmannulidae	Dysteriina	Dysteriidae	
*Hartmannula*	Dysteriidae	*Dysteria*	
Dysteriidae	*Dysteria*	*Trochilia*	
*Dysteria*	Trochiliidae	Hartmannulidae	
*Trochilia*	*Trochilia*	*Hartmannula*	
Chonotrichida		Chonotrichia	
Exogemmina		Exogemmida	
Chilodochonidae		Chilodochonidae	
*Chilodochona*		*Chilodochona*	

The subclasses Cyrtophoria, Rhynchodia, Chonotrichia, and Suctoria each constitutes a clade within the monophyletic class Phyllopharyngea (de Puytorac, 1994; Lynn and Small, 1997; Lynn, 2008; Table 1). The chonotrichians, which are sessile symbionts typically found on the mouthparts of crustaceans, have long been recognized as phyllopharyngeans (Corliss, 1979; Lynn, 2016). Previous studies have revealed strong similarities between the cortical microtubular components of chonotrichians (represented by *Chilodochona*) with the cortical components of free-living cyrtophorians (Dobrzaprnska-Kaczanowska, 1963; Grain and Batisse, 1974; Fahrni, 1982). However, molecular phylogenies for chonotrichians were lacking (Gao et al., 2012, 2014). Cyrtophorians are characterized by a glabrous dorsal side and prominent cytopharyngeal basket composed of a large number of microtubules (Lynn, 2008). Although several cyrtophorians have been studied recently (Chen et al., 2016; Pan et al., 2016; Zhang et al., 2018), the systematics of this group remains ambiguous. Therefore, the class Phyllopharyngea needs to be re-evaluated based on more data. In the present study, genomic and transcriptomic data were used to analyze the evolutionary relationships among the synhymenians, nassophoreans, and phyllopharyngeans.

## Materials and Methods

### Single-Cell Sample Preparation

Six freshwater ciliate species (*Chilodontopsis depressa*, *Trithigmostoma cucullulus*, *Trochilia petrani*, *Trochilia* sp., *Dysteria derouxi*, and *Hartmannula sinica*) were collected from Xiaoxihu Lake, Qingdao, China (120.35°E, 36.06°N). For each species, cells were washed in phosphate-buffered saline (PBS) buffer (without Mg^2+^ or Ca^2+^), and genomic DNA extracted from a single cell was amplified using a Single Cell WGA Kit (Yikon, YK001A) based on MALBAC technology (Lu et al., 2012). The RNA of *C. depressa* and *D. derouxi* was extracted from a single cell using the RNeasy kit (Qiagen, Hilden, Germany) and digested with DNase. The rRNA fraction was depleted using GeneRead rRNA Depletion Kit (Qiagen, Hilden, Germany). The raw data of single-cell genome sequencing of *Chilodochona* sp. were supplied by Dr. Denis H. Lynn (University of British Columbia, Vancouver, Canada).

### High-Throughput Sequencing and Data Processing

Illumina libraries of 300 bp were prepared from amplified single-cell genomic DNA using Nextera DNA Flex Library Prep Kit by random PCR with primers (Illumina #20018704, United States) according to manufacturer’s instructions. Paired-end sequencing (150 bp read length) was performed using an Illumina HiSeq4000 sequencer. The sequencing adapter was trimmed and low quality reads (reads containing >10% Ns or 50% bases with *Q*-value < = 5) were filtered out by Trimmomatic v0.36 (using parameters: LEADING:3 TRAILING:3 SLIDINGWINDOW:4:15 MINLEN:36) (Bolger et al., 2014).

Single-cell genomes of the seven species were assembled using SPAdes v3.11.1 (default parameters and −*k* = 21, 33, 55, 77) (Bankevich et al., 2012; Nurk et al., 2013). Mitochondrial genomic peptides of ciliates along with bacterial and human genome sequences were downloaded from NCBI GenBank as BLAST databases to remove contamination caused by mitochondria, bacteria, or factitious sources (BLAST + v2.6.0, *E*-value cutoff = 1*e*−5). CD-HIT v4.7 (CD-HIT-EST, -c 0.98 -n 10 -r 1) was employed to eliminate the redundancy of contigs (with sequence identity threshold = 98%) (Fu et al., 2012). Poorly supported contigs (coverage < 1 and length < 400 bp) were discarded by a custom Perl script, considering the inherent characteristics of the single-cell genomic sequencing technique (Zong et al., 2012). QUAST v4.6.1 was used to evaluated the resulting contigs (−*M* = 0) (Gurevich et al., 2013).

For the three nassophorean species, *Nassula variabilis*, *Furgasonia blochmanni*, and *Pseudomicrothorax dubius*, RNA sequences were obtained from the published dataset (NCBI accession number PRJNA434361). The adapters of reads were trimmed by Trimmomatic v0.36 (parameters as described above), and the clean reads were used for transcriptome assembly by Trinity v2.6.6 (default parameters) (Grabherr et al., 2011). Any redundancy and contamination of transcriptome assemblies were removed by BLAST and CD-HIT, as described above.

RNA-seq transcriptome data for another 21 ciliate species were downloaded from the Marine Microbial Eukaryote Transcriptome Sequencing Project^1^ plus six ciliate species whose high-quality data were acquired from public databases (data sources are summarized in Supplementary Table S1).

### Intron Length Distribution and Stop Codon Preference Detection

RNA-seq data of *C. depressa* were mapped to the corresponding reference genome assembly by HISAT2 v2.1.0 (Kim et al., 2015) and the transcriptome was assembled by StringTie v1.3.4d (Pertea et al., 2016). The size distribution of introns was analyzed by custom Perl scripts and the intronic boundary motif was visualized by WebLogo3 (Crooks et al., 2004). The frequency of stop codon usage was estimated by a custom Perl script which recognized the three nucleotides following the codon of the last amino acid in each matched complete protein sequences. This is identified as a potential stop codon by BLAST (*E*-value ≤ 1*e*−10, identity ≥ 30%, alignment length ≥ 50 amino acids) transcript sequences of investigated species against ciliate protein database in Uniprot^2^ (detail of method was uploaded to the GitHub repository: Bryan0425/Stop_codon_usage). Among different groups of ciliates, Student’s *t*-test was performed in inner-class and outer-class.

### Gene Prediction and Annotation

Transcriptomes were analyzed with TransDecoder v5.1.0^3^ (*Tetrahymena* genetic codes for *N. variabilis*, universal genetic codes for the other three species, *F. blochmanni*, *P. dubius*, and *C. depressa*) to search the longest ORFs, which generated protein predictions longer than 100 amino acids. To validate the potential ORFs, the predicted protein sequences were blasted to the Swiss-Prot database (*E*-value < 1*e*−5) (UniProt, 2018) and matched to the Pfam-A database (El-Gebali et al., 2018) by HMMER v3.1b2 (Eddy, 2009). Confirmed protein products were further annotated using four databases (CDD, Pfam, SUPERFAMILY, and TIGRFAM) by InterProScan v5.29 (Mitchell et al., 2018), and the ciliate gene database from NCBI GenBank by BLAST + v2.6.0 (*E*-value cutoff < 1*e*−5). RepeatMasker v4.0.7 (Smit, 1996) was employed to annotate non-coding RNA and repeat sequences. *De novo* gene prediction was performed by AUGUSTUS v3.3 (–species = tetrahymena, rearranging only TGA as stop codon, TAA/TAG as Glutamine) (Keller et al., 2011).

### Ortholog Detection and GO Pathway

Orthologs among *C. depressa* and other ciliates were detected by a reciprocal BLAST hit (RBH) approach (*E*-value < 1*e*−5, identity ≥ 30%, alignment length ≥ 50 aa) and pairwise mutual best-hits were identified as putative orthologs. A Venn diagram was generated by the R package VennDiagram (Chen and Boutros, 2011). Gene Ontology (GO) term enrichment analysis was performed by BiNGO v3.0.3 (p.adjust < 0.05) (Maere et al., 2005), which was integrated in Cytoscape v3.4.0 (Shannon et al., 2003). The bubble plots were generated by the R package ggplot2 (Wickham, 2016).

### Datasets and Alignments

Newly characterized sequences that combined relevant sequences obtained from the GenBank database were assembled into two datasets: (1) phylogenomics data (59 taxa in total), i.e., the 2 newly sequenced trascriptomes; 5 newly sequenced genomes; and 39 other ciliates, plus 9 apicomplexans and 4 dinoflagellates as outgroups; (2) SSU-rDNA database (56 taxa in total), i.e., 5 newly sequenced genes plus 3 heterotrichs and karyorelictids as outgroups. Sequences information and GenBank accession numbers are shown in Supplementary Table S2.

### Phylogenetic and Phylogenomic Analyses

Small subunit ribosomal DNA sequences were multiple-aligned and trimmed to be blunt using BioEdit 7.1.3.0 (ClustalW algorithm) with the default parameters. Maximum-likelihood (ML) and Bayesian inference (BI) analyses were carried out on the online server CIPRES Science Gateway (Miller et al., 2010; Penn et al., 2010), using RAxML-HPC2 on XSEDE v8.2.10 (Stamatakis, 2014) with GTRGAMMA model and MrBayes on XSEDE v3.2.6 (Ronquist et al., 2012) with GAMMA model calculated by MrModeltest v2.3 (Nylander, 2004), respectively. In the ML analyses, 1,000 bootstraps were performed to assess the reliability of internal branches. For Bayesian analyses, Markov chain Monte Carlo (MCMC) simulations were run with two sets of four chains for 4,000,000 generations, with sampling every 100 generations and a burn-in of 10,000 (Chen et al., 2015). All the remaining trees were used to calculate posterior probabilities (PP) using a majority rule consensus. MEGA v7.0.20 (Kumar et al., 2016) was used to visualize the tree topologies.

The phylogenomic analyses were carried out by the GPSit pipeline (using parameters -e 1e-10 -d 50 -g 100000 -f 100 relaxed masking mode) based on 157 orthologs shared by all 46 ciliates utilizing a “supermatrix” approach (Chen et al., 2018b). The multiple sequence alignment was uploaded to CIPRES Science Gateway. RAxML-HPC2 v8.2.9 (using parameters: LG model of amino acid substitution + Γ distribution + F, four rate categories, 500 bootstrap replicates) and PHYLOBAYES MPI 1.5a (using parameters: CAT-GTR model + Γ distribution, four independent chains, 10,000 generations for matrix of relaxed masking, or 20,000 generations for matrix of stringent mode, with first 10% of all generations as burn-in, convergence Maxdiff < 0.3) were performed to generate the ML and BI trees, respectively (Le and Gascuel, 2008; Lartillot et al., 2009; Miller et al., 2010; Stamatakis, 2014; Gentekaki et al., 2017). All trees were visualized by MEGA v7.0.20. To visualize all available phylogenetic signals in the supermatrix, phylogenetic network analyses were calculated with SPLITSTREE v4.14.4 (using parameters: Network = NeighborNet; 1000 bootstrap replicates) (Huson and Bryant, 2005).

## Results

### Morphology of Synhymenians, Nassophoreans, and Phyllopharyngeans

**FIGURE 2:**
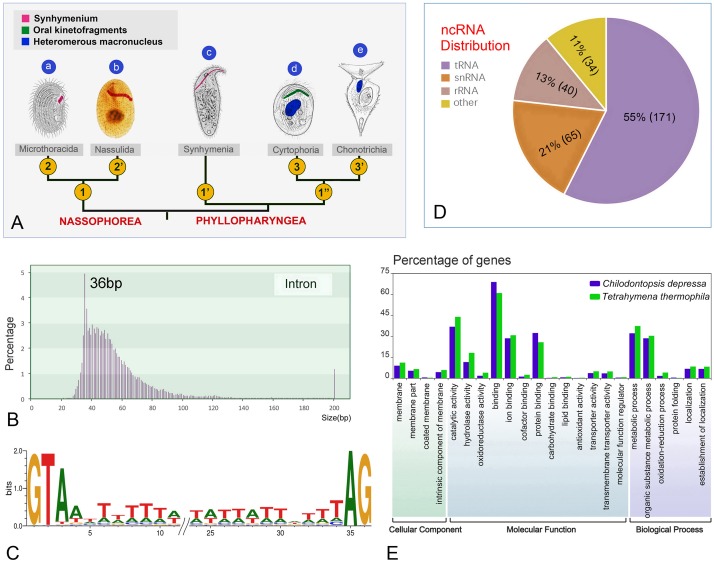
*Chilodontopsis depressa* morphology and draft genome profile. **(A)** Cladogram showing the evolutionary relationships within the Synhymeniida, Nassophorea, and Phyllopharyngea based on morphological data. Parts **(a–d)** are original and part **(e)** is from Jankowski (1973). 1, Synhymenium restricted on left ventral sometimes dorsal surface; 1′, synhymenium extends from left to right ventral surface; 1^″^, synhymenium absent; 2, synhymenium usually comprises three adoral polykinetids; 2′, synhymenium comprises more than three adoral polykinetids; 3, free-living, merotelokinetal division, oral kinetofragments present; 3′, sessile, division by budding, oral kinetofragments absent. **(B)** Bar chart showing the distribution of intron sizes calculated by customized Perl script. Pink bars represent percentage of introns frequency. Introns over 200 bp in length were integrated in the right bar. **(C)** Combining genome and transcriptome sequencing identified 5′-GT-AG-3′ as the representative motif with the third conserved A site by WebLogo3. **(D)** Pie chart showing the proportions of different types of non-coding RNA predicted and calculated by RepeatMask annotation: tRNA (purple), snRNA (orange), rRNA (brown), and other kind (yellow). **(E)** The whole genome was annotated using Interproscan and Gene Ontology (GO) enrichment analysis was performed by WEGO of *C. depressa* (blue) and *T. thermophila* (green). Bar chart showing the percentages of gene numbers.

The main morphological characteristics of the synhymenians, nassophoreans, and phyllopharyngeans are summarized in Figure 2A. Their divergence/convergence has been described in detail and discussed previously (Hausmann and Peck, 1978; Eisler, 1988; Chen et al., 2018a). Briefly, synhymenians and nassophoreans have a synhymenium, which is a kinetic structure below the oral apparatus, mainly on the ventral surface. In synhymenians the synhymenium extends from left to right on the ventral surface, but in Nassophorea it is restricted to the left ventral surface. This serves as the main characteristic to distinguish these two groups. With the exception of the synhymenians, this structure is absent in the Phyllopharyngea.

### Genomic Features of Classes Phyllopharyngea and Nassophorea

Although the newly sequenced ciliates have differing genome sizes and GC contents, most genome assemblies of phyllopharyngeans are about 40 M (Table 2). *Chilodontopsis depressa* has small, AT-rich introns, most of which are 36 bp (Figure 2B) with a GT-AG motif and a conservative third A site (Figure 2C). Also, of the non-coding RNA types predicted by RepeatMasker, 55% are tRNAs (171), 21% are snRNAs (65), 13% are rRNAs (40), and 11% are other RNAs (34) (Figure 2D). The predicted genes were annotated according to cellular component, molecular function, and biological process (Figure 2E and Supplementary Figure S2). Compared to *Tetrahymena thermophila*, *C. depressa* has a high gene content in its binding and protein-binding pathways, which might be associated with the molecular function of its well-developed cytopharyngeal basket of microtubules and its algivorous mode of nutrition (Tucker, 1968; Weisenberg et al., 1968; Chaaban and Brouhard, 2017).

**TABLE 2:** Information of genome assembly from single-cell sequencing.

**Statistics**	***Chilodontopsis depressa***	***Trithigmostoma cucullulus***	***Trochilia petrani***	***Trochilia* sp.**	***Chilodochona* sp.**	***Dysteria derouxi***	***Hartmannula sinica***
Assembly size (bp)	129,702,864	48,714,649	41,378,687	119,916,114	45,682,069	46,996,486	41,739,095
Number of contigs	89,876	32,789	22,283	73,516	38,938	23,348	18,986
Largest contig	36,898	27,797	56,445	93,598	37,703	40,963	57,867
N50 (bp)	2,084	2,143	4,035	3,260	1,568	3,365	4,393
GC (%)	34.23	51.62	38.14	42.89	40.05	44.33	42.87

**FIGURE 3:**
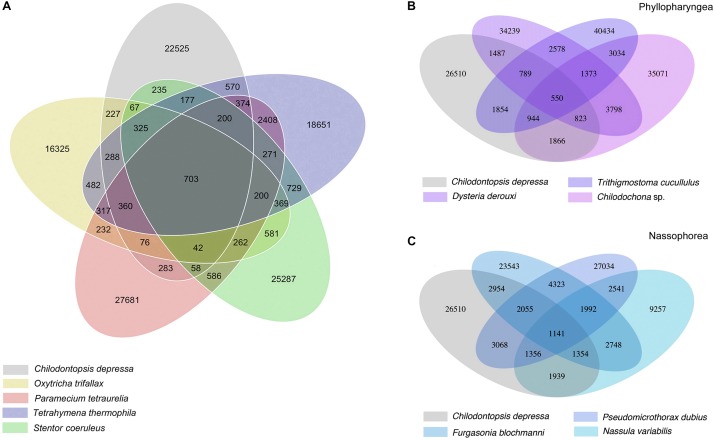
Venn diagram showing ortholog detection among *Chilodontopsis depressa*, phyllopharyngeans, nassophoreans, and other model ciliates. **(A)** Venn diagram showing the comparison of numbers of orthologous genes among *C. depressa* (gray) and other typical ciliate species, including *Tetrahymena thermophila* (blue), *Paramecium tetraurelia* (pink), *Oxytricha trifallax* (yellow), and *Stentor coeruleus* (green), calculated by R package. **(B)** Venn diagram showing the comparison of numbers of orthologous genes among *C. depressa* and phyllopharyngean ciliates, including *Trithigmostoma cucullulus*, *Dysteria derouxi*, and *Chilodochona* sp. **(C)** Venn diagram showing the comparison of numbers of orthologous genes among *C. depressa* and nassophorean ciliates, including *Nassula variabilis*, *Furgasonia blochmanni*, and *Pseudomicrothorax dubius.*

**FIGURE 4:**
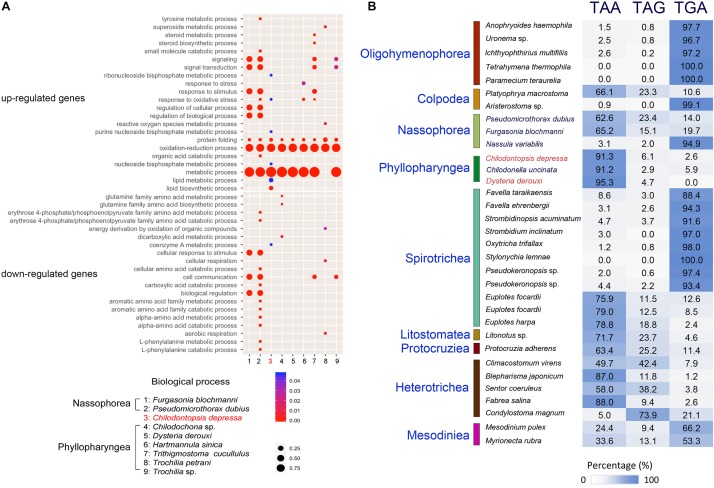
Comparative genomic analysis of *Chilodontopsis depressa* and phyllopharyngean and nassophorean ciliates. **(A)** Bubble plot showing comparison of genes controlling biological process among nassophorean (*Furgasonia blochmanni*, *Pseudomicrothorax dubius*) (1–2), synhymenian (*C. depressa*) (3), and phyllopharyngean (*Chilodochona* sp., *Dysteria derouxi*, *Hartmannula sinica*, *Trithigmostoma cucullulus*, *Trochilia petrani*, and *Trochilia* sp.) (4–9) ciliates based on Gene Ontology analysis by R package ggplot2. The upper and lower panels show the pathways in which up-regulated and down-regulated genes are enriched, respectively. The color bar and dot size measure the *p*-values of the gene enrichment and the percentage of covered genes in the corresponding pathways, respectively. **(B)** Bar chart showing the bias of stop codon usage among *C. depressa*, phyllopharyngeans, nassophoreans, and other well-annotated ciliates.

Orthologous genes were identified among the seven species sequenced in the current work and other previously sequenced ciliates (Figure 3 and Supplementary Figure S1). 703 orthologous genes shared by *Chilodontopsis depressa* and four model ciliates were identified (Figure 3A). *C. depressa* shared fewer orthologous genes with phyllopharyngeans (550) than with nassophoreans (1141), which is consistent with its closer morphological resemblance to the latter, e.g., the presence (vs. absence) of a synhymenium (Figures 2A, 3B,C). Moreover, the GO term enrichment results suggested that *C. depressa* shared more metabolic features with phyllopharyngeans than with nassophoreans (Figure 4A and Supplementary Figure S2), such as regulation of cellular processes, regulation of biological processes, cellular response to stimulus, and biological regulation pathways. Such features tend to be enriched in nassophoreans not phyllopharyngeans.

Based on the homolog search between transcripts of *C. depressa* and protein sequences of other ciliates, the most frequently used stop codon in *C. depressa* was identified as TAA (91.3%, Figure 4B). Compared with model ciliates such as *T. thermophila* and *Oxytricha trifallax*, stop codon usages of other newly sequenced phyllopharyngeans and nassophoreans were also predicted. The results showed that the stop codon usage of *C. depressa* was closer to *Chilodonella uncinata* and *D. derouxi* (both of which belong to Phyllopharyngea) (deviation = 1.9) than to Nassophorea species (deviation = 25.6). The preference of stop codon usage was well-conserved within each class (*p*-value = 0.01538 < 0.05 by *t*-test).

### Phylogenetic and Phylogenomic Analyses

**FIGURE 5:**
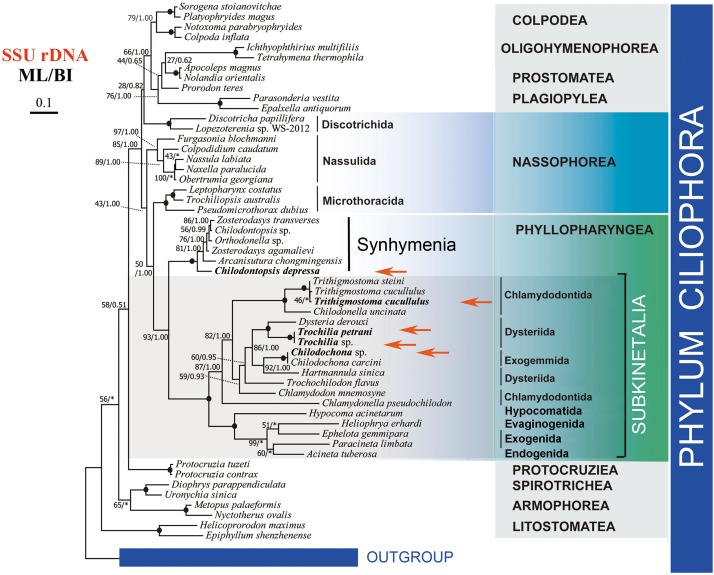
Phylogenetic tree based on the SSU rDNA sequence data. The five newly sequenced species are shown in bold and with red arrows. Numbers at nodes represent bootstrap values of ML from 1000 replicates and the posterior probability of BI, respectively. Clades with a different topology between the ML and BI tree are indicated by “^∗^”. Scale bar corresponds to 10 substitutions per 100 nucleotide positions.

The SSU rDNA dataset provided the most abundant taxon sampling. We carried out phylogenetic analyses based on 56 SSU-rDNA sequences (Figure 5). In the resulting trees, *C. depressa* grouped with the typical synhymenians, albeit in the basal position. The subclass Synhymenia grouped with the class Phyllopharyngea (or “Subkinetalia,” a name coined for the class Phyllopharyngea with synhymenians excluded), with high support values (ML/BI, 93/1.00). The monophyly of Subkinetalia is fully supported. The class Nassophorea is paraphyletic in the SSU rDNA tree, with the three orders (Microthoracida, Nassulida, and Discotrichida) separating to various positions within the CONthreeP assemblage (a robust clade comprising the classes Colpodea, Oligohymenophorea, Nassophorea, Prostomatea, Plagiopylea, and Phyllopharyngea) (Lynn, 2008; Adl et al., 2019).

**FIGURE 6:**
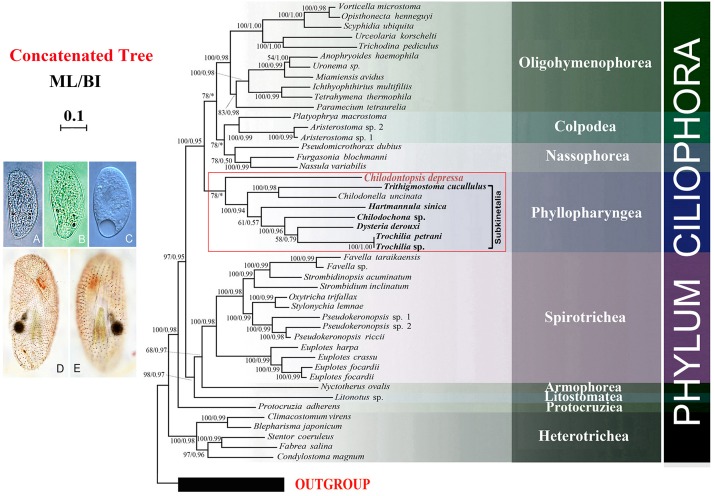
Phylogenomic tree concatenated from ML and BI analyses. Newly sequenced taxa are shown in bold. Photomicrographs of *Chilodontopsis depressa* from life **(A–C)** and after silver natrate staining **(D,E)**. Numbers at nodes are BI posterior probability followed by ML bootstrap values. The scale bar corresponds to 0.1 expected substitutions per site. Clades with a different topology between the ML and BI tree are indicated by “^∗^”.

Seven new genomes and two new transcriptomes were generated in the present study. The “omics” data source of orthologous genes for phylogenomic analyses has thus been increased to 46 ciliates, although *C. depressa* remains the only representative synhymenian (Figure 6). By using GPSit with relaxed mode (Chen et al., 2018b), we established a database with 157 genes comprising 173,835 amino acid residues. In the resulting phylogenomic tree using the ML algorithm, *C. depressa* clustered with the Subkinetalia with moderate support (ML, 78). The monophyly of the Subkinetalia was strongly supported by both ML and BI analyses. The branching pattern within Subkinetalia is in accordance to the SSU rDNA phylogeny inferences (Figure 5), although present taxon sampling is restricted to the subclass Cyrtophoria. For the class Nassophorea, transcriptome data are available for three species representing two orders, i.e., Nassulida and Microthoracida. The three species grouped together with a moderate support (ML/BI, 78/0.50). The classes Nassophorea and Colpodea formed a moderately supported clade in the ML tree, which subsequently grouped with the class Oligohymenophorea. The class Phyllopharyngea was sister to the Colpodea + Nassophorea + Oligohymenophorea clade according to the ML algorithm.

## Discussion

### The Systematic Assignment of Synhymenians–Nassophoreans–Phyllopharyngeans Assemblage

The systematic position of synhymenians has long puzzled taxonomists, and their assignment to the class Nassophorea (nassulids and microthoracids) or to the Subkinetalia have been controversial. Synhymenians share ultrastructural and morphological similarities with both Nassophorea and Phyllopharyngea, suggesting that they are likely a transition group between these two classes (Eisler and Bardele, 1983; Small and Lynn, 1985; Kivimaki et al., 1997; Lynn and Small, 2002). A comprehensive comparison of morphology and ultrastructure among synhymenians, Nassophorea, and the Subkinetalia has previously addressed this issue (Gong et al., 2009). The SSU rDNA phylogeny (Figure 5) and previous studies support a close relationship between synhymenians and Subkinetalia, and thus synhymenians were suggested as a subclass (Synhymenia) within the class Phyllopharyngea (Zhang et al., 2014; Gao et al., 2016, 2017; Liao et al., 2018).

In this study we provide genomic data for a synhymenian, i.e., *Chilodontopsis depressa*, for the first time. *C. depressa* occupied the basal position within the subclass Synhymenia (Figure 5), indicating that this species might be an ideal representative to demonstrate the ancestral candidate of the synhymenians. The phylogenomic analysis moderately supported (ML, 78) the affiliation of *C. depressa* to Subkinetalia, while the nassophoreans (represented by nassulids and microthoracids) formed another moderately supported monophyletic group (Figure 6). This is consistent with findings of previous studies based on SSU rDNA and multi-gene data which inferred that synhymenians are more closely related to Phyllopharyngea than to Nassophorea (Gong et al., 2009; Gao et al., 2016).

It is noteworthy that bootstrap support values for grouping *C. depressa* with Subkinetalia are lower in the phylogenomic analysis than those based on SSU rDNA data (ML, 78 vs. ML/BI, 93/1.00), whereas support for the monophyly of Subkinetalia are high in both analyses. These findings imply that, on a genomic scale, synhymenians and Subkinetalia are more divergent than indicated by the SSU rDNA gene (Figures 5, 6). This is consistent with the obvious ultrastructural differences between these two groups: in synhymenians, the postciliary microtubules of each kinetosome form a double row (vs. triads in cyrtophorians), and the synhymenian monokinetids lack both the subkinetal ribbons of microtubules and the dense transverse fibrils that characterize the phyllopharyngeans (Kivimaki et al., 1997). Moreover, in Subkinetalia, there are some extremely specialized Subkinetalian groups, such as the suctorians and chonotrichians (Figure 2A), the adults of which are sessile and divide by budding. Collectively, these data indicate that there was a long history, and that a number of evolutionary events occurred, after the synhymenians and Subkinetalia diverged from their common ancestor.

The phylogenomic tree recovered only low to moderate support for the monophyly of Nassophorea (i.e., orders Nassulida and Microthoracida, with Discotrichida excluded). This is in accordance to a recent phylogenomic study of the nassulids (Lynn et al., 2018) and multi-gene analyses of the major ciliate lineages (Gao et al., 2016). The cytopharyngeal basket of nassulids is composed of one or more out of three sets of microtubular lamellae, namely cytostomal (Z), subcytostomal (Y), and nematodesmal (X) lamellae (Hausmann and Peck, 1978; de Puytorac and Njiné, 1980; Eisler, 1988). Microthoracids (e.g., *Pseudomicrothorax* and *Leptopharynx*) have only X lamella (Hausmann and Peck, 1978; de Puytorac and Njiné, 1980; Eisler, 1988), synhymenians have only Z lamella (Kivimaki et al., 1997), and cyrtophorians have only Z and Y lamellae (Kurth and Bardele, 2001). Controversial scenarios have been proposed to deduce the evolution of the cytopharyngeal basket apparatus based on phylogenetic (Gong et al., 2009) and phylogenomic analyses (Lynn et al., 2018). Inclusion of the new genomic data for the synhymenians in the analyses results in partial support for the previous hypothesis: synhymenians and Subkinetalia are members of the class Phyllopharyngea not Nassophorea (Gong et al., 2009; Gao et al., 2016). According to a scenario of morphological evolution deduced by Lynn et al. (2018), if the present phylogenomic tree is a probable representation of the order of divergence of these groups, i.e., a cytopharyngeal basket with microtubular nematodesmata and with only Z microtubular ribbons would probably be an ancestral feature of the CONthreeP main clade. A loss of Z lamella might have happened in the microthoracids, while the X and Y lamellae in nassophoreans and phyllopharyngeans could be apomorphies acquired later to enable the rapid ingestion of different food resources (Tucker, 1968; Hausmann and Peck, 1978; Lynn et al., 2018).

Nowadays, SSU rDNA is a powerful phylogenetic marker to resolve many evolutionary relationships, although it has its limitations (Hasegawa and Hashimoto, 1993). For example, SSU rDNA sequences that are similar in nucleotide composition might have been placed incorrectly close together in phylogenetic trees (Woese et al., 1991). In addition, because of random variation of single genes, it is risky to infer the phylogeny from any single gene. Thus, it is suggested that single gene phylogenetic analyses should be corroborated by use of other phylogenetic markers (Ludwig and Klenk, 2001). Following the development of illumina sequencing, phylogenomic analysis is used as the gold standard to estimate evolutionary relationships on the “genome level” (Brown et al., 2001; Ciccarelli et al., 2006; Wu and Eisen, 2008). Phylogenomic data are usually expected to be well resolved without many limitations of small data sets, e.g., relatively less informative characters from independent loci and random noise (Delsuc et al., 2005; Jeffroy et al., 2006). Among the present analysis, 157 proteins sequences of 59 species were used to constructed the phylogenomic tree. Therefore, a more robust estimation of evolutionary relationships could be obtained. On the other hand, our results show that on a genomic scale, there are more variations indicated than those on single gene phylogenetic analysis, like SSU rDNA (Figures 5, 6). Phylogenomic analysis applies much more data from whole genome/transcriptome compared to a specific one gene. Furthermore, proteins sequences exhibit more differences and variations among species when used to as a material of analysis. Some statistical support values in the tree were relatively high, but some were low or not as high as that in single-gene phylogenetic analysis, e.g., 58/0.79 versus full value at the branch of *Dysteria* and *Trochilia*. Higher support values might be achieved if the number of informative sites is inadequate or the key species are not included in the analysis. Furthermore, gene selection of phylogenomic analysis is an important process. The orthologs must to be considered in analysis, especially phylogenetic markers such as *HSP70* and tubulin (Baldauf et al., 2000; Ludwig and Klenk, 2001).

### Tiny Introns Characterize Ciliate Genomes as Class Level

All eukaryotic genomes that have been sequenced harbor introns, but so far none have been identified in prokaryotes. Intron size varies in genomes of different species, e.g., from 169 bp in the model flowering plant *Arabidopsis thaliana* to 7,386 kb on average in humans (Jo and Choi, 2015; Piovesan et al., 2015). Previous studies of human genomes have suggested that intron size may play an important role in governing alternative splicing and sensing self/foreign circular RNAs (Fox-Walsh et al., 2005; Dewey et al., 2006; Kim et al., 2006; Chen et al., 2017). Furthermore, the minimum length of introns required for the splicing reaction and other advanced biological functions is thought to be 30 bp (Roy et al., 2008; Piovesan et al., 2015). However, smaller introns (15 bp) have been reported in heterotrich ciliates (e.g., *Stentor coeruleus* and *Condylostoma magnum*) (Slabodnick et al., 2017). Tiny introns have also been identified in the classes Armophorea (*Nyctotherus ovalis*: 27 bp) (McGrath et al., 2007; Ricard et al., 2008), Spirotrichea (*O. trifallax*: 38 bp) (Swart et al., 2013), and Oligohymenophorea (*T. thermophila*: 74 bp and *Paramecium* species: 26 bp) (Swart et al., 2013; Arnaiz et al., 2017). The present study reports for the first time tiny introns (36 bp) in the genome of species of the class Phyllopharyngea (Figures 2A,B). Thus, based on the present and previous studies, tiny introns are another prevalent important feature of ciliate genomes just like the variation of rDNA copy numbers (Wang et al., 2019). Whether the small size of these introns impair their biological functions, governing alternative splicing for instance, requires further exploration.

### Stop Codon Usage in Different Groups of Ciliates

Stop codon preference in the terminal region has been intensively investigated by evolutionary biologists (Alff-Steinberger and Epstein, 1994; Smith and Smith, 1996; Jungreis et al., 2011). Previous studies have reported the flexibility of the nuclear genetic code in ciliates by demonstrating that standard stop codons are reassigned to amino acids (Lozupone et al., 2001; Heaphy et al., 2016; Swart et al., 2016). One possible reason is that ambiguous genetic codes enabled the ancestors of ciliates to thrive during a certain periods in their evolutionary history (Swart et al., 2016). To determine how the stop codon rearrangement is conserved in different lineages, we systematically assessed the preference of stop codon usage in 33 ciliates based on their transcriptome data (Figure 4B). Our results show that stop codon usage varies among different classes and, in some cases, within a class. For example, the stop codon usage of *C. depressa* (formerly classified in the class Nassophorea, order Synhymeniida) is more similar to that of *C. uncinata* (class Phyllopharyngea, order Chlamydodontida) and *D. derouxi* (class Phyllopharyngea, order Dysteriida) than to nassophoreans (e.g., *N. variabilis*, *P. dubius*, and *F. blochmanni*), suggesting Synhymeniida and other phyllopharyngeans may have a common recent ancestor. The close relationship between Synhymeniida and Phyllopharyngea is also supported by the results of phylogenomic analysis (Figure 6). We therefore posit that the preference of stop codon usage can be adopted as an extra parameter to resolve phylogenetic relationships. However, how the preference of stop codon usage is inherited remains poorly understood and should be addressed in future studies.

## Data Availability Statement

All Illumina sequencing datasets are deposited in the NCBI under the Accession Number PRJNA546036.

## Author Contributions

MM, BP, and XC conceived the study. BP and XC analyzed the data and interpreted the results using bioinformatics methods. LH, QZ, and ZQ performed the phylogenetics analysis and morphology identification and description. AW revised the writing of the manuscript. All authors contributed to the manuscript and agreed on the manuscript before review.

## Conflict of Interest

The authors declare that the research was conducted in the absence of any commercial or financial relationships that could be construed as a potential conflict of interest.

## Footnotes

1 https://www.imicrobe.us

2 https://www.uniprot.org/

3 http://transdecoder.github.io/

## Supplementary Material

FIGURE S1 Molecular information of *Chilodontopsis depressa* and phyllopharyngean ciliates. Distribution of species with best hits by BLAST. The X-axis represents percentage of contigs or genes. **(A)** *Hartmannula sinica*, **(B)** *Chilodochona* sp., **(C)** *Dysteria deroux*i, **(D)** *Trithigmostoma cucullulus*, **(E)** *Trochilia petrani*, **(F)** *Trochilia* sp., and **(G)** *Chilodontopsis depressa*.

FIGURE S2 Comparative genomic analysis of *Chilodontopsis depressa* and phyllopharyngean and nassophorean ciliates. Bubble plot showing comparison of genes controlling cell component **(A)** and molecular function **(B)** among nassophorean (*Furgasonia blochmanni*, *Pseudomicrothorax dubius*) (1,2), synhymenian (*Chilodontopsis depressa*) (3), and phyllopharyngean (*Chilodochona* sp., *Dysteria derouxi*, *Hartmannula sinica*, *Trithigmostoma cucullulus*, *Trochilia petrani* and *Trochilia* sp.) (4–9) ciliates based on Gene Ontology analysis by R package ggplot2.
