# Supplementary figures and images for: Comparative Genomics Analysis of Ciliates Provides Insights on the Evolutionary History Within “Nassophorea–Synhymenia–Phyllopharyngea” Assemblage
Source: Front Microbiol. 2019 Dec 12;10:2819. doi: 10.3389/fmicb.2019.02819 (PMC6920121; doi:10.3389/fmicb.2019.02819)

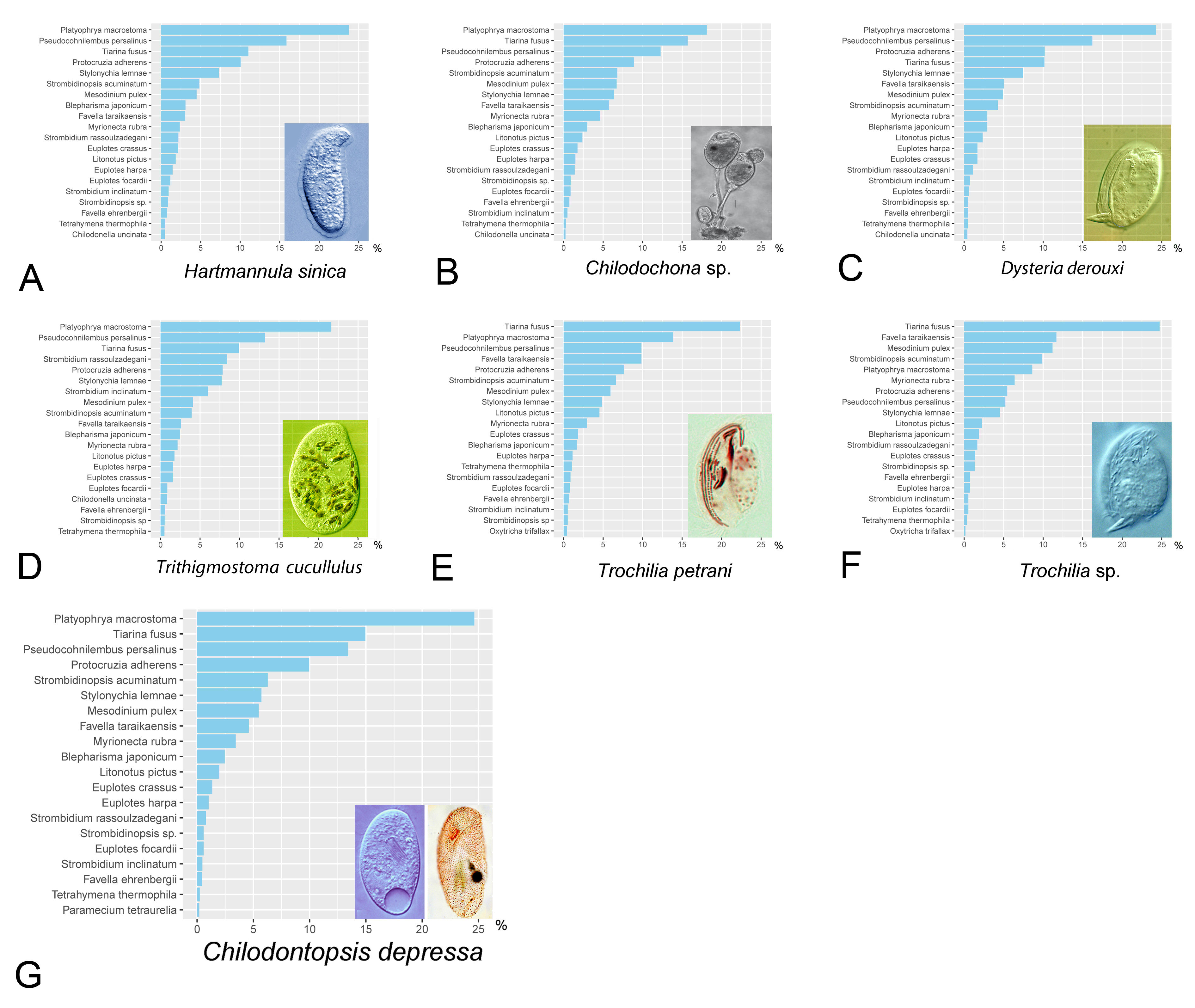

Supplement: FIGURE S1 — Molecular information of Chilodontopsis depressa and phyllopharyngean ciliates. Distribution of species with best hits by BLAST. The X-axis represents percentage of contigs or genes. (A) Hartmannula sinica, (B) Chilodochona sp., (C) Dysteria derouxi, (D) Trithigmostoma cucullulus, (E) Trochilia petrani, (F) Trochilia sp., and (G) Chilodontopsis depressa. [file Image_1.jpg]

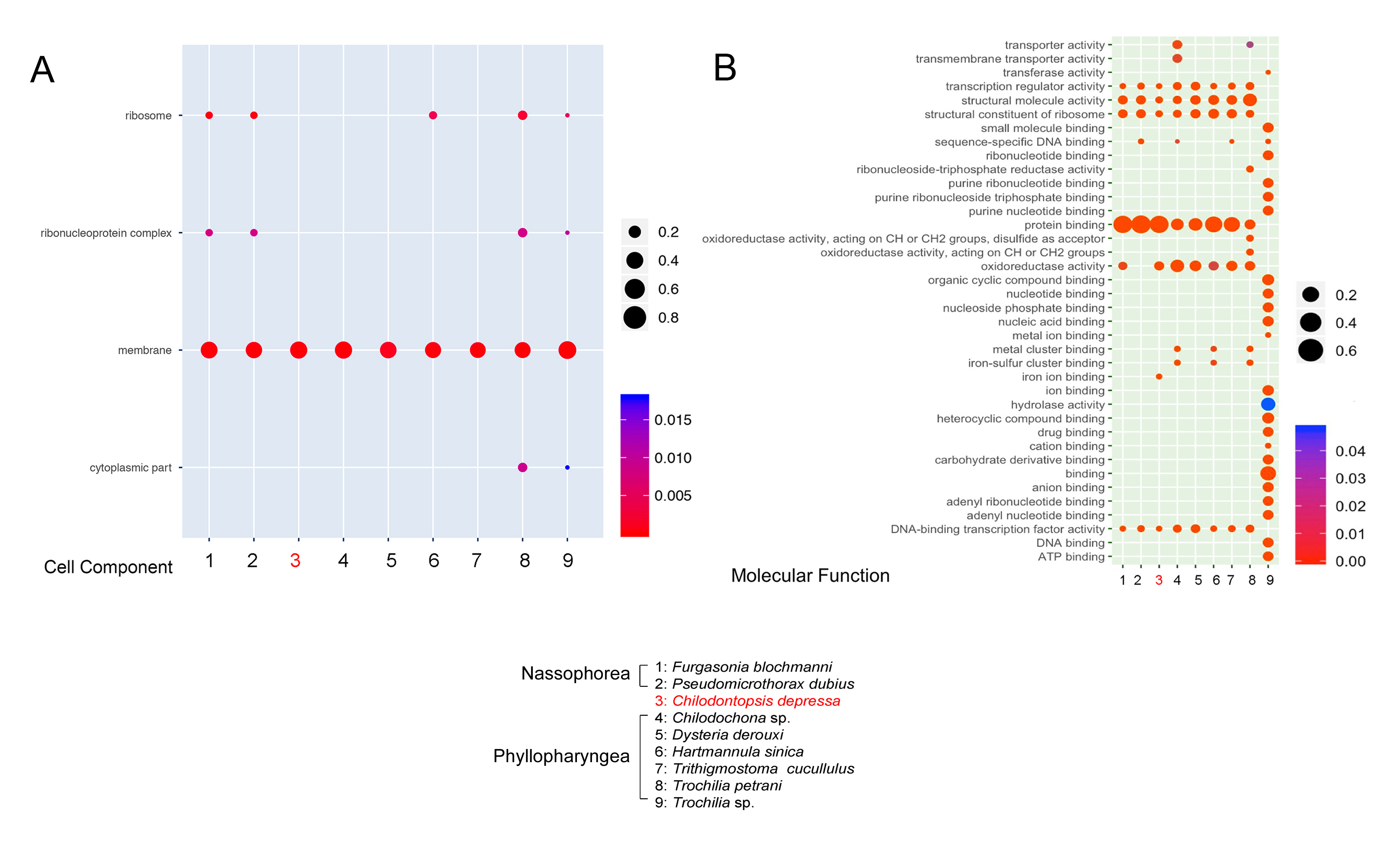

Supplement: FIGURE S2 — Comparative genomic analysis of Chilodontopsis depressa and phyllopharyngean and nassophorean ciliates. Bubble plot showing comparison of genes controlling cell component (A) and molecular function (B) among nassophorean (Furgasonia blochmanni, Pseudomicrothorax dubius) (1,2), synhymenian (Chilodontopsis depressa) (3), and phyllopharyngean (Chilodochona sp., Dysteria derouxi, Hartmannula sinica, Trithigmostoma cucullulus, Trochilia petrani and Trochilia sp.) (4–9) ciliates based on Gene Ontology analysis by R package ggplot2. [file Image_2.jpg]
