# Supplementary material for: Comparative Genomics Analysis of Ciliates Provides Insights on the Evolutionary History Within “Nassophorea–Synhymenia–Phyllopharyngea” Assemblage
Source: Front Microbiol. 2019 Dec 12;10:2819. doi: 10.3389/fmicb.2019.02819 (PMC6920121; doi:10.3389/fmicb.2019.02819)
Supplement: Supplementary file 3 [file Table_1.DOC]

**Supplemental table 1.**

**List of the transcriptome data available in the present work.**

|  | **Species** | **Taxonomy** | **No.** | **Website** |
| --- | --- | --- | --- | --- |
|  | ***Anophryoides haemophila*** | Oligohymenophorea | MMETSP1018 | [https://www.imicrobe.us](https://www.imicrobe.us/) |
|  | ***Aristerostoma* sp.** | Colpodea | MMETSP0125 |  |
|  | ***Blepharisma japonicum*** | Heterotrichea | MMETSP1395 |  |
|  | ***Climacostomum virens*** | Heterotrichea | MMETSP1397 |  |
|  | ***Condylostoma magnum*** | Heterotrichea | MMETSP0210 |  |
|  | ***Euplotes focardii*** | Spirotrichea | MMETSP0205 |  |
|  | ***Euplotes focardii*** | Spirotrichea | MMETSP0206 |  |
|  | ***Euplotes harpa*** | Spirotrichea | MMETSP0213 |  |
|  | ***Fabrea salina*** | Heterotrichea | MMETSP1345 |  |
|  | ***Favella ehrenbergii*** | Spirotrichea | MMETSP0123 |  |
|  | ***Favella taraikaensis*** | Spirotrichea | MMETSP0434 |  |
|  | ***Litonotus* sp.** | Litostomatea | MMETSP0209 |  |
|  | ***Mesodinium pulex*** | Mesodiniea | MMETSP0467 |  |
|  | ***Myrionecta rubra*** | Mesodiniea | MMETSP0798 |  |
|  | ***Platyophrya macrostoma*** | Colpodea | MMETSP0127 |  |
|  | ***Protocruzia adherens*** | Protocruziea | MMETSP0216 |  |
|  | ***Pseudokeronopsis* sp.** | Spirotrichea | MMETSP0211 |  |
|  | ***Pseudokeronopsis* sp.** | Spirotrichea | MMETSP1396 |  |
|  | ***Strombidinopsis acuminatum*** | Spirotrichea | MMETSP0126 |  |
|  | ***Strombidium inclinatum*** | Spirotrichea | MMETSP0208 |  |
|  | ***Uronema* sp.** | Oligohymenophorea | MMETSP0018 |  |
|  | ***Ichthyophthirius multifiliis*** | Oligohymenophorea |  | http://ich.ciliate.org/ |
|  | ***Oxytricha trifallax*** | Spirotrichea |  | http://oxy.ciliate.org/ |
|  | ***Paramecium teraurelia*** | Oligohymenophorea |  | http://ciliate.org/ |
|  | ***Sentor coeruleus*** | Heterotrichea |  | http://stentor.ciliate.org/ |
|  | ***Stylonychia lemnae*** | Spirotrichea |  | http://stylo.ciliate.org/ |
|  | ***Tetrahymena thermophila*** | Oligohymenophorea |  | http://ciliate.org |
|  | ***Chilodonella uncinata*** | Phyllopharyngea | PRJNA161607 |  |
