# Supplementary material for: Comparative Genomics Analysis of Ciliates Provides Insights on the Evolutionary History Within “Nassophorea–Synhymenia–Phyllopharyngea” Assemblage
Source: Front Microbiol. 2019 Dec 12;10:2819. doi: 10.3389/fmicb.2019.02819 (PMC6920121; doi:10.3389/fmicb.2019.02819)
Supplement: Supplementary file 4 [file Table_2.DOC]

**Supplemental table 2.**

**List of species for which SSU rDNA, 5.8S rDNA, LSU rDNA were used in the present work. New sequences were shown as “*” which were obtained from our omics data.**

|  | **Class** | **Order** | **Taxon** | **18S** |
| --- | --- | --- | --- | --- |
|  | COLPODEA | Bryophryida | ***Notoxoma parabryophryides*** | EU039903 |
|  | COLPODEA | Colpodida | ***Colpoda inflata*** | KM222106 |
|  | COLPODEA | [Sorogenida](https://www.ncbi.nlm.nih.gov/Taxonomy/Browser/wwwtax.cgi?mode=Undef&id=164619&lvl=3&keep=1&srchmode=1&unlock) | ***Sorogena stoianovitchae*** | AF300285 |
|  | COLPODEA | [Cyrtolophosidida](https://www.ncbi.nlm.nih.gov/Taxonomy/Browser/wwwtax.cgi?mode=Undef&id=88560&lvl=3&keep=1&srchmode=1&unlock) | ***Platyophryides magus*** | HM140395 |
|  | NASSOPHOREA | Discotrichida | ***Discotricha papillifera*** | JQ918368 |
|  | NASSOPHOREA | Microthoracida | ***Lopezoterenia* sp.** | JQ918369 |
|  | NASSOPHOREA | Microthoracida | ***Leptopharynx costatus*** | EU286811 |
|  | NASSOPHOREA | Microthoracida | ***Trochiliopsis australis*** | JQ918367 |
|  | NASSOPHOREA | Microthoracida | ***Pseudomicrothorax dubius*** | FM201298 |
|  | NASSOPHOREA | Nassulida | ***Colpodidium caudatum*** | EU264560 |
|  | NASSOPHOREA | Nassulida | ***Furgasonia blochmanni*** | X65150.1 |
|  | NASSOPHOREA | Nassulida | ***Nassula labiata*** | KC832949 |
|  | NASSOPHOREA | Nassulida | ***Naxella paralucida*** | KY652918 |
|  | NASSOPHOREA | Nassulida | ***Obertrumia georgiana*** | X65149 |
|  | OLIGOHYMENOPHOREA | Ophryoglenida | ***Ichthyophthirius multifiliis*** | U17354 |
|  | OLIGOHYMENOPHOREA | Tetrahymenida | ***Tetrahymena thermophila*** | X56165 |
|  | PHYLLOPHARYNGEA | Chlamydodontida | ***Trithigmostoma cucullulus*** | FJ998037 |
|  | PHYLLOPHARYNGEA | Chlamydodontida | ***Trithigmostoma cucullulus*** | ***** |
|  | PHYLLOPHARYNGEA | Chlamydodontida | ***Chlamydodon mnemosyne*** | FJ998031 |
|  | PHYLLOPHARYNGEA | Chlamydodontida | ***Chlamydonella pseudochilodon*** | FJ998032 |
|  | PHYLLOPHARYNGEA | Dysteriida | ***Dysteria derouxi*** | KM222105 |
|  | PHYLLOPHARYNGEA | Dysteriida | ***Trochochilodon flavus*** | JN867018 |
|  | PHYLLOPHARYNGEA | Dysteriida | ***Hartmannula sinica*** | EF623827.1 |
|  | PHYLLOPHARYNGEA | Hypocomatida | ***Hypocoma acinetarum*** | JN867019 |
|  | PHYLLOPHARYNGEA | Endogenida | ***Acineta tuberosa*** | FJ865206 |
|  | PHYLLOPHARYNGEA | Evaginogenida | ***Heliophrya erhardi*** | AY007446 |
|  | PHYLLOPHARYNGEA | Exogenida | ***Ephelota gemmipara*** | EU600180 |
|  | PHYLLOPHARYNGEA | Exogenida | ***Paracineta limbata*** | FJ865207 |
|  | PHYLLOPHARYNGEA | Synhymeniida | ***Orthodonella* sp.** | KC832952 |
|  | PHYLLOPHARYNGEA | Synhymeniida | ***Zosterodasys agamalievi*** | FJ008926 |
|  | PHYLLOPHARYNGEA | Synhymeniida | ***Chilodontopsis depressa*** | ***** |
|  | PHYLLOPHARYNGEA | Synhymeniida | ***Chilodontopsis* sp.** | FJ998040 |
|  | PHYLLOPHARYNGEA | Synhymeniida | ***Zosterodasys transverses*** | EU286812.1 |
|  | PHYLLOPHARYNGEA | Synhymeniida | ***Arcanisutura chongmingensis*** | KY652917.1 |
|  | PHYLLOPHARYNGEA | Chlamydodontida | ***Trithigmostoma steini*** | X71134.1 |
|  | PHYLLOPHARYNGEA | Chlamydodontida | ***Chilodonella uncinata*** | KY476314.1 |
|  | PHYLLOPHARYNGEA | Dysteriida | ***Trochilia petrani*** | JN867016.1 |
|  | PHYLLOPHARYNGEA | Dysteriida | ***Trochilia* sp.** | ***** |
|  | PHYLLOPHARYNGEA | Exogemmida | ***Chilodonchona* sp*.*** | ***** |
|  | PHYLLOPHARYNGEA | Exogemmida | ***Chilodonchona carcini*** | KU588417.1 |
|  | PLAGIOPYLEA | Odontostomatida | ***Epalxella antiquorum*** | EF014286.1 |
|  | PLAGIOPYLEA | Plagiopylida | ***Parasonderia vestita*** | JN857941.1 |
|  | PROSTOMATEA | Prorodontida | ***Nolandia orientalis*** | KM222103 |
|  | PROSTOMATEA | [Prorodontida](https://www.ncbi.nlm.nih.gov/Taxonomy/Browser/wwwtax.cgi?mode=Undef&id=6001&lvl=3&keep=1&srchmode=1&unlock) | ***prorodon teres*** | X71140 |
|  | PROSTOMATEA | Colepadie | ***Apocoleps cf. magnus*** | FJ858213.1 |
|  | PROTOCRUZIEA | Protocruziida | ***Protocruzia contrax*** | DQ190467 |
|  | PROTOCRUZIEA | Protocruziida | ***Protocruzia tuzeti*** | KU500620.1 |
|  | ARMOPHOREA | Armophorida | ***Metopus palaeformis*** | AY007450 |
|  | ARMOPHOREA | Clevelandellida | ***Nyctotherus ovalis*** | AJ222678 |
|  | HETEROTRICHEA | Heterotrichida | ***Fabrea salina*** | KM222110 |
|  | HETEROTRICHEA | Heterotrichida | ***Condylostoma magnum*** | KM222108 |
|  | KARYORELICTEA | Protostomatida | ***Kentrophoros gracilis*** | FJ467506 |
|  | LITOSTOMATEA | Haptorida | ***Helicoprorodon maximus*** | KM222102 |
|  | LITOSTOMATEA | Pleurostomatida | ***Epiphyllum shenzhenense*** | GU574809 |
|  | SPIROTRICHEA | Euplotida | ***Diophrys parappendiculata*** | EU267928 |
|  | SPIROTRICHEA | Euplotida | ***Uronychia sinica*** | FJ876982 |
